# Sensorimotor Cortical Neuroplasticity in the Early Stage of Bell's Palsy

**DOI:** 10.1155/2017/8796239

**Published:** 2017-02-19

**Authors:** Wenwen Song, Minhui Dai, Lihua Xuan, Zhijian Cao, Sisi Zhou, Courtney Lang, Kun Lv, Maosheng Xu, Jian Kong

**Affiliations:** ^1^The First Affiliated Hospital of Zhejiang Chinese Medical University, Hangzhou, China; ^2^Department of Psychiatry, Massachusetts General Hospital and Harvard Medical School, Charlestown, MA 02129, USA

## Abstract

Neuroplasticity is a common phenomenon in the human brain following nerve injury. It is defined as the brain's ability to reorganize by creating new neural pathways in order to adapt to change. Here, we use task-related and resting-state fMRI to investigate neuroplasticity in the primary sensory (S1) and motor cortex (M1) in patients with acute Bell's palsy (BP). We found that the period directly following the onset of BP (less than 14 days) is associated with significant decreases in regional homogeneity (ReHo), fractional amplitude of low frequency fluctuations (fALFF), and intrinsic connectivity contrast (ICC) values in the contralateral S1/M1 and in ReHo and ICC values in the ipsilateral S1/M1, compared to healthy controls. The regions with decreased ReHo, fALFF, and ICC values were in both the face and hand region of S1/M1 as indicated by resting-state fMRI but not task-related fMRI. Our results suggest that the early stages of BP are associated with functional neuroplasticity in both the face and hand regions of S1/M1 and that resting-state functional fMRI may be a sensitive tool to detect these early stages of plasticity in patient populations.

## 1. Introduction

Neuroplasticity is a common, yet complex phenomenon that occurs in both animals and humans [1–4]. In neural injury patients, this plasticity can play a significant role in the development of disease [5]. For example, following a stroke, the ipsilateral cortex becomes the dominant cortex used to control the affected limb, but with treatment there is a shift back to contralateral control [6, 7]. Amputation patients are another example of cortical map changes following a profound injury [8–10]. These neural injury models have provided investigators a chance to investigate neural plasticity in the human brain, and have significantly enhanced our understanding of the development of neural plasticity. However, the complexities of amputation and stroke studies, including variations in affected locations, duration of the injury, and accompanied psychiatric comorbidities, have limited the interpretation of the observed results.

Bell's palsy (BP) offers an ideal human model to investigate neuroplasticity following peripheral nerve injury [11–20]. BP is the paralysis of the unilateral facial expression muscles caused by the reactivation of the herpes virus. This reactivation impairs the signaling from the motor cortex to the affected facial muscle. Compared to other conditions such as stroke and amputation, BP is associated with fewer complications and fewer or no medications, allowing us to explore the development of cortical reorganization directly following peripheral nerve injury.

The primary somatosensory cortex (S1) and the primary motor cortex (M1) are well known for their precise physical representation of the body on the cortex, thereby providing a perfect location to explore the reorganization of the brain caused by BP. Brain imaging tools such as MRI/fMRI, MEG, and PET and neuromodulation methods have allowed investigators to explore changes in the brain's structure, function, and excitability at S1 and M1 following different disorders [21–27].

Recently, intrinsic resting-state functional connectivity (rsFC) has drawn the attention of investigators, and been applied to investigate neural plasticity [28, 29]. Different methods have been developed to investigate the resting-state functional connectivity. Some methods such as seed-based analysis and independent component analysis, focus on the networks changes (between/among different brain regions). Other methods, such as regional homogeneity (ReHo), intrinsic connectivity contrast (ICC), and fractional amplitude of low frequency fluctuations (fALFF), focus on regional brain functional status. ReHo is a measure of the time similarity of a given voxel to its nearest 26 voxels [30]. fALFF is used to characterize the regional, spontaneous neuronal activity [31, 32], while ICC tests the strength of the global connectivity pattern between each voxel and the rest of the brain [33]. Although each of these methods focus on different characteristics of resting-state functional connectivity, all of these methods may help us target key regions, rather than networks, providing valuable tools to study neuroplasticity in S1 and M1 in BP patients.

In this study we first used task-related fMRI (hand movement and mouth movement) to define subject's cortical map at S1 and M1 and then investigated and compared the regional brain functional status in the early period of BP to healthy controls using ReHo [30], fALFF [31, 32], and ICC [33]. Our objective was to find the acute functional influence of peripheral nerve injury on the central nervous system.

## 2. Method

### 2.1. Subjects

Twenty-five right-handed patients with left or right side Bell's palsy (House-Brackmann Scale (HBS) ≥3, age 36 ± 7 years ranging from 23 to 50, 15 males, 10 females, 14 right side facial paralysis) were recruited from the acupuncture department at the First Affiliated Hospital of Zhejiang Chinese Medical University. All patients with Bell's palsy onset less than 14 days underwent the MRI scan [11]. We recruited 25 age- and gender-matched healthy controls. No study participants had a history of physical or psychological disorders. The study was approved by the Ethics Committee at the First Affiliated Hospital of Zhejiang Chinese Medical University.

### 2.2. MRI Experimental Design

**Figure 1:**
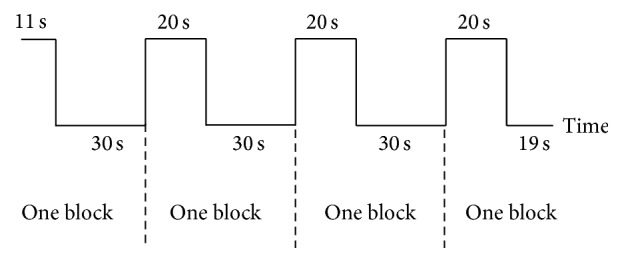
The block design paradigm used in this study.

There were a total of 5 tasks in our design: a facial movement task, left and right hand movement tasks, and left and right facial sensory stimulation task. We used a block design in our 5 tasks (Figure 1). The frequency of the movement or sensory stimulation was 13–16 times per 20 seconds. In the facial movement task, subjects were asked to purse their lips. In the hand movement tasks, we asked subjects to open and close their right or left hand. In the facial sensory tasks, we brushed subjects' right or left jaw. Prior to being scanned, all participants underwent practice trials to ensure the mastery of these exercises. Each task-related fMRI scan contained 4 blocks. The instructions were explained orally. All patients and healthy controls finished the hand movement and facial sensory tasks, and 21 healthy controls finished the facial movement task. The BP patients were not required to perform the facial movements due to their inability to perform facial expressions.

### 2.3. MRI Imaging Acquisition

All scans were performed on the same 3.0 Tesla MR scanner (Magnetom Verio, Siemens, Germany) in order to obtain T1-weighted structural images and echo-planar T2^*∗*^-weighted images (EPI). Structural images were obtained by MP-RAGE sequence: TR = 1900 ms, TE = 2.45 ms, FA = 9 degrees, voxel size = 1*∗*1*∗*1 mm, matrix = 256*∗*256. Two hundred time points of functional resting-state data were acquired by EPI session: TR = 2000 ms, TE = 30 ms, FA = 90 degrees, slices = 33, and voxel size = 4.0 × 4.0 × 4.0 mm at the beginning of the MRI scan, while subjects kept their eyes opened. Sixty time points of functional task-state data were obtained by EPI sequence: TR = 3000 ms, TE = 30 ms, FA = 90 degrees, slices = 36, voxel size = 3.2 × 3.2 × 3.0 mm, and gap = 0.75 mm.

### 2.4. fMRI Data Analysis

All functional data preprocessing and analyses were performed using Data Processing Assistant for Resting-State fMRI (DPARSF) software (http://rfmri.org/DPARSF) [34] and CONN toolbox v15.g [35] (http://www.nitrc.org/projects/conn) in MATLAB (MathWorks, Inc., Natick, Massachusetts). DPARSF is based on Statistical Parametric Mapping (SPM8) (http://www.fil.ion.ucl.ac.uk/spm) and a Resting-State fMRI Data Analysis Toolkit (http://www.restfmri.net) [36]. All statistical analyses were performed by SPM8.

Before we processed the images, all BP patients with left side palsy and matched healthy controls were flipped along the *y*-axis (R-L flip) so their images could be directly compared to the patients with right side palsy [14]. Given the important role of S1 and M1 in neural plasticity changes following a nerve injury [10, 37–41], we defined the bilateral pre- and postcentral gyrus as the region of interest (ROI), and made a mask based on automated anatomical labeling (AAL) [42].

### 2.5. Task Data Analysis

For each subject, all images were realigned using a six-parameter rigid-body transformation that corrected for motion artifacts. The images were then coregistered with the subjects' corresponding anatomical (T1-weighted) images, normalized by using structural images unified segmentation, resampled to 3 mm cubic voxels, and spatially smoothed with a 6 mm full width at half maximum (FWHM) Gaussian kernel.

A multiple regression analysis using a general linear model was performed to obtain statistical parametric maps calculated for all 5 conditions (facial movement, right/left side hand movement and right/left side facial sensory). Functional MRI signal time courses were high-pass filtered (128 s) and modeled as an experimental stimulus onset function, and convolved by the canonical hemodynamic response function (low-pass filter). Individual contrast images for each task were entered into a 2nd level random effects analysis to make inferences at the group level. For our one sample* t*-test comparing facial movement, hand movement, and facial sensory stimulation with baseline, a threshold of voxel-wise *p* < 0.05 FWE corrected was applied. For between group comparisons (BP versus controls), we applied a threshold of *p* < 0.005 and small volume FWE corrected *p* < 0.05 in ROI regions as defined above. A threshold of voxel-wise *p* < 0.005 uncorrected and cluster-level *p* < 0.05 family wise error (FWE) correction was applied to non ROI brain regions.

### 2.6. Resting-State Data Analysis

For each subject, the first 10 volumes of functional data were discarded because of the signal equilibrium and subjects' adaptation to the imaging noise. The remaining volumes were then slice timing corrected, spatially realigned, and coregistered with the subject's corresponding anatomical (T1-weighted) images and segmented. Friston 24 head motion parameters [43, 44] and CSF signals were regressed out in ReHo and fALFF data. All images were normalized using structural unified segmentation and resampled to 3 mm cubic voxels, and then ReHo data was filtered using the low frequency band (0.01–0.08 Hz). Frames with FD 0.2 mm (“scrubbing”) were removed. Individuals with 50% of their time series removed were excluded from the analyses. After scrubbing, no one was excluded from the analysis. We did a ReHo analysis by calculating the Kendall's coefficient of concordance (KCC) of a given voxel with those of its nearest neighbors (26 voxels) in a voxel-wise analysis [30]. The ReHo data was then spatially smoothed with a 6 mm FWHM Gaussian kernel.

fALFF data was smoothed after normalization, and then, temporal filtering was performed so that only the low frequency band (0.01–0.08) was examined in subsequent analyses of the low frequency fluctuations (LFF) amplitude.

After normalization, the ICC data underwent ART (http://www.nitrc.org/projects/artifact_detect) to eliminate correlations caused by head motion and artifacts, by identifying outlier time points in the motion parameters and global signal intensity. For each subject, we treated images as outliers if composite movement from a preceding image exceeded 0.5 mm, or if the global mean intensity was >3 SDs from the mean image intensity for the entire resting scan. ICC analysis [33] was carried out by applying a voxel-voxel approach using the CONN toolbox v15.g. This analysis determined the strength of functional connectivity between each voxel and all other voxels in the brain. The ICC data was then smoothed with a 6 mm FWHM Gaussian kernel.

To explore the ReHo/fALFF/ICC differences between the BP patient group and healthy control group, a two-sample* t*-test was performed on ReHo/fALFF/ICC z-maps. For between group comparisons (BP versus controls), we applied a threshold of *p* < 0.005 and small volume FWE corrected *p* < 0.05 in the ROI as defined above. A threshold of voxel-wise *p* < 0.005 uncorrected and cluster-level *p* < 0.05 family wise error (FWE) correction was applied to non ROI brain regions.

## 3. Results

### 3.1. Behavioral Results

All patients showed impairments in their left or right facial muscle function due to BP, with the HBS grades ≥3. All MR scans were performed 3–14 days after BP onset (Table 1).

### 3.2. Task-Related fMRI Results

We found significantly increased activation at the bilateral S1/M1, supplementary motor area (SMA), and cerebellum in healthy controls during the facial movement task.

During the hand movement task, we found significantly increased activation at the contralateral S1/M1, thalamus, putamen, insula, bilateral SMA, and cerebellum in both BP patients and healthy controls. There was no significant difference between the two groups.

During facial sensory stimulation, we found significantly increased activation at the contralateral S1/M1, insula and S2 in both BP patients and healthy controls. We also found that in the healthy control group the (1) left facial sensory stimulation provoked significantly increased activation at the ipsilateral S1 and the (2) right facial sensory stimulation provoked significantly increases activation at the ipsilateral cerebellum (Table 2). There was no significant difference between the two groups.

### 3.3. Resting-State Functional Connectivity Results

Compared to healthy controls, BP patients showed (1) significant ReHo decreases in the bilateral S1/M1 and contralateral cingulate motor area (CMA), (2) significant fALFF decreases in the contralateral S1/M1, and (3) significant ICC decreases in the bilateral S1/M1 (Table 3, Figures 3 and 4).

**Figure 2:**
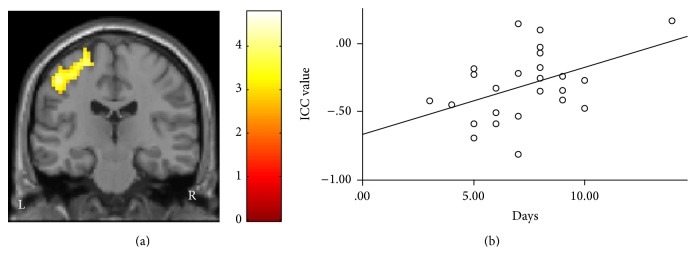
Intrinsic connectivity contrast (ICC) results. (a) ICC value was decreased in the contralateral S1/M1 in Bell's palsy patients. (b) There was a positive association between the duration of a subject's BP and the ICC value (*p* < 0.035).

**Figure 3:**
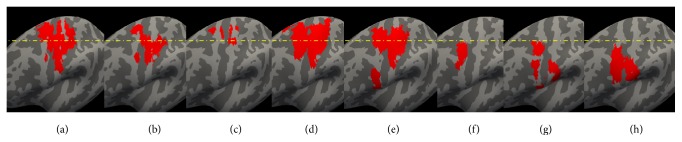
Contralateral hemisphere to the side of the affected nerve injury. (a) ReHo resting-state fMRI result. (b) ICC resting-state fMRI result. (c) fALFF resting-state fMRI result. (d) Increased activation evoked by ipsilateral hand movement in BP patients. (e) Increased activation evoked by ipsilateral hand movement in healthy controls. (f) Increased activation evoked by ipsilateral facial sensory stimulation in BP patients. (g) Increased activation evoked by ipsilateral facial sensory stimulation in healthy controls. (h) Increased activation evoked by mouth movement in healthy controls.

**Figure 4:**
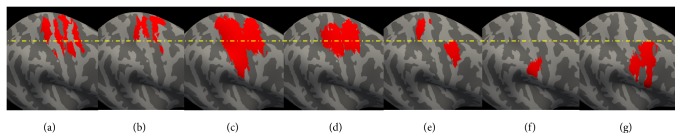
Ipsilateral hemisphere to the side of the affected nerve injury. (a) ReHo resting-state fMRI result. (b) ICC resting-state fMRI result. (c) Increased activation evoked by contralateral hand movement in BP patients. (d) Increased activation evoked by contralateral hand movement in healthy controls. (e) Increased activation evoked by contralateral facial sensory stimulation in BP patients. (f) Increased activation evoked by contralateral facial sensory stimulation in healthy controls. (g) Increased activation evoked by mouth movement in healthy controls.

To explore the association between the above measurements and the duration of the patients' Bell's palsy, we also performed regression analyses across all patients using SPSS 18.0 Software (SPSS Inc., Chicago, IL, USA), including age, gender, and HBS as covariates. We found a significant positive association between the ICC values of the contralateral S1/M1 and the duration of the patient's BP (*p* = 0.035) (Figure 2).

## 4. Discussion

In this study, we explored neural plasticity in BP patients using task-related and resting-state fMRI. We found that the early stage of BP is associated with significant decreases in ReHo, fALFF and ICC values in the contralateral S1/M1, and with significant decreases in ReHo and ICC values in the ipsilateral S1/M1, compared to matched healthy controls. It is interesting to note that the regions with decreased ReHo, fALLFF and ICC values were not only in the cortical map representing the face, but also in the hand region of the cortex; regions defined by hand and mouth task-related fMRI. We also found that BP patients and healthy controls had similar increased activation during the hand movement task and facial sensory stimulation. There was no significant difference between the two groups in all tasks performed by both groups. Our results suggest functional neuroplasticity in S1/M1 following the onset of BP and that resting-state fMRI may be a sensitive tool to detect these early stages of neural plasticity in patient populations.

ReHo, ICC, and fALFF are methods used to investigate the regional brain functional status using resting-state fMRI. ReHo measures the homogeneity of a brain's functional area in a specific condition, and has the ability to detect unpredicted hemodynamic responses that model-driven methods have failed to identify [30]. ICC tests the connectivity strength between each voxel and the rest of the brain [33]. fALFF measures regional, spontaneous neuronal activity, and can significantly suppress nonspecific signal components in resting-state MRI and increase sensitivity to regional spontaneous brain activity [32]. Although each of these methods focus on different aspects of brain function/activity, all of these methods focus on specific brain regions, rather than the networks, allowing us to target specific brain regions associated with the neuropathology of disorders.

In a previous study, investigators compared the resting-state functional connectivity of the motor network as identified by facial movements. They found that compared to healthy controls, BP patients showed decreased connectivity, mainly in areas responsible for sensorimotor integration and supervision (SII, insula, thalamus and cerebellum) [14]. In a more recent study, investigators found that the resting-state functional connectivity between the ipsilateral ACC and other brain regions such as the contralateral M1, SMA, ipsilateral S2, and mid-cingulate cortex increased with increased duration (ranging from 2–55 days) of BP [12]. In another study, investigators used the hand region of bilateral S1 as a seed to investigate the connectivity before and after acupuncture treatment at the contralateral side of the hand in BP patients. They found resting-state functional connectivity between S1 and bilateral S1, S2, and precuneus was significantly decreased in BP patients shortly following nerve injury [11].

In our study, we used methods focused on the regional brain status, and found significant decreases in resting-state functional connectivity at the S1/M1. This is consistent with previous studies on nerve injury. For instance, Pawela and colleagues found that the contralateral S1 showed a decreased connectivity with the bilateral M1 and ipsilateral S1 in rats with transected the brachial plexus compared to the control rats [45]. In another study, Makin and colleagues found the resting-state functional connectivity was decreased between the contralateral and ipsilateral hand regions in S1/M1 in upper-limb amputees compared to the healthy controls [22, 29].

We found decreased ReHo, fALFF, and ICC values not only at face representation areas, but also at hand representation areas of S1 and M1. These results are consistent with previous studies on neuroplasticity following peripheral nerve injury, in which researchers found the preservation of the original function of the invaded territory, as well as cohabitation with a newly acquired function [29, 46].

We also found a significant decrease in ReHo and ICC values at the ipsilateral S1/M1, suggesting that unilateral nerve injury can affect bilateral S1/M1. Studies have suggested that the ipsilateral hemisphere also plays an important role in sensorimotor function. For instance, a TMS study showed that stimulation to one side of the M1 cortex can cause ipsilateral motor evoked potentials (MEPs) in BP patients [20]. In amputation patients, imagining the movement of the phantom limb can evoke activation of bilateral S1/M1 [9, 47].

We found there was a significant positive association between BP duration and ICC values at the contralateral S1 and M1. We speculate this may reflect the self-recovery process of BP patients, which usually begins about one week after the onset of BP. Further studies are needed to validate the above findings.

In this study, we found that hand movement evoked significantly increased activation at bilateral M1, SMA, and insula, which is consistent with findings from previous studies [14, 15]. In an early PET study on facial palsy patients, Rijntjes et al. found that finger tapping tasks provoked increased regional cerebral blood flow at the region of S1/M1 representing the hand and face in the all eight patients with facial palsy duration ranging from 29 days to 36 years compared to controls, but not in a patient with a duration of 7 days [18]. In another study, Klingner and colleagues found there was no significant difference in fMRI results between BP patients and healthy controls during a mouth movement task [14]. These results are consistent with our findings that there is not any significant difference in fMRI results between the acute stage of BP when patients are compared to healthy controls in the sensory and motor tasks.

The present study had several limitations. The first is that we did not scan the patients at late stage or after recovery, so we were not able to provide dynamic neural plasticity changes of BP patients. Second, we did not test the facial motor condition in our BP patients, therefore we were unable to measure the motor cortical function during this task and compare it to the healthy controls. However, a previous study found no significant difference between BP patients and healthy controls during a mouth movement task [14]. Finally, we used the ICC method, which measures both positive and negative functional connectivity. Positive and negative connectivity may be associated with different physiological meanings, but both represent valuable information. We would like to emphasize that this method has been successfully used in many other studies [48–53]. We therefore believe that ICC is an appropriate method to apply to this study.

## 5. Conclusion

We found that the period shortly following the onset of BP is associated with neural plasticity in both the face and hand region of the bilateral S1/M1 as measured by different regional functional connectivity methods. Our results imply that regional functional connectivity may be a useful tool to investigate neural plasticity in patient populations and may hold the potential to assess the severity of the injured nerve.

## Figures and Tables

**Table 1:** Clinical data for all BP patients.

Number	Gender	Age (years)	Paralyzed side	Duration (days)	HBS
1	M	44	R	7	4
2	M	42	R	10	3
3	M	38	R	7	3
4	M	25	L	14	5
5	M	39	R	7	4
6	M	34	L	9	4
7	F	34	R	5	4
8	F	34	R	6	5
9	F	23	R	5	4
10	M	28	L	8	5
11	M	40	L	8	5
12	M	33	R	7	5
13	F	39	L	4	5
14	M	39	L	6	5
15	F	45	L	6	4
16	F	33	R	3	4
17	M	26	L	9	4
18	M	26	R	10	4
19	M	43	R	8	5
20	F	35	L	8	6
21	M	42	R	9	6
22	M	31	R	5	6
23	F	50	L	8	4
24	F	39	L	8	6
25	F	34	R	5	4

F, female; HBS, House-Brackmann Scale; L, left side; M, male; R, right side.

**Table 2:** Task-related fMRI results for healthy controls.

Tasks	Brain region	Cluster size	Peak *Z* score	MNI coordinates (mm)		
				*x*	*y*	*z*
Facial movement	L S1/M1	346	7.38	−51	−12	42
	R S1/M1	273	6.65	48	−9	45
	R SMA	175	6.42	6	−3	63
	B cerebellum	126	6.34	12	−63	−18

Right hand movement	L S1/M1	1216	7.58	−33	−24	54
	B cerebellum	505	7.27	12	−54	−15
	L thalamus	535	7.01	−15	−21	6

Left hand movement	R S1/M1	533	7.50	36	−21	51
	L cerebellum	552	7.14	−18	−54	−18
	B SMA	434	6.96	6	0	45
	R thalamus	531	6.75	15	−21	6
	R putamen		6.02	24	3	6
	R S2	149	6.05	48	−21	24

Right facial sensory	L S2/S1/M1	744	6.84	−39	−15	18
	R cerebellum	42	5.84	6	−66	−12

Left facial sensory	R S2/S1/M1	201	6.74	60	−18	24
	L S1	29	5.47	−60	−18	30

B, bilateral; L, left; M1, primary motor cortex; R, right; S1, primary somatosensory cortex; S2, secondary somatosensory cortex; SMA, supplementary motor area.

**Table 3:** Comparisons of ReHo, fALFF, and ICC values in resting-state between BP patients and healthy controls using small volume FWE correction.

Resting-state	Brain region	Cluster size	Peak *Z* score	MNI coordinates (mm)		
				*x*	*y*	*z*
ReHo	L S1/M1	500	4.59	−33	−24	63
	R S1/M1	234	4.09	24	−36	60
fALFF	L S1/M1	40	3.36	−30	−27	63
ICC	L S1/M1	224	4.33	−48	−18	48
	R S1/M1	140	4.60	15	−21	69

B, bilateral; L, left; M1, primary motor cortex; R, right; S1, primary somatosensory cortex.
